# Role of clinical pharmacists in palliative care team: A scoping review

**DOI:** 10.1017/S1478951525101545

**Published:** 2026-01-13

**Authors:** Sen Li, Qin Wang, Benling Qi, Lijuan Bai, Jiaqiang Xu, Haiying Sun, Yihui Liu

**Affiliations:** 1 Department of Pharmacy, Union Hospital, Tongji Medical College, Huazhong University of Science and Technology, Wuhan, China; 2 Department of Radiology, Union Hospital, Tongji Medical College, Huazhong University of Science and Technology, Wuhan, China; 3 Department of Geriatrics, Union Hospital, Tongji Medical College, Huazhong University of Science and Technology, Wuhan, China; 4 Department of Otorhinolaryngology, Union Hospital, Tongji Medical College, Huazhong University of Science and Technology, Wuhan, China

**Keywords:** Clinical pharmacist, palliative care, multidisciplinary team, pharmaceutical service, non-communicable diseases

## Abstract

**Objectives:**

Clinical pharmacists are increasingly recognized as essential members of multidisciplinary palliative care teams, yet their specific roles and impact have not been comprehensively summarized. This scoping review aimed to systematically map and synthesize published evidence on the clinical roles, interventions, and professional contributions of pharmacists within multidisciplinary palliative care services for patients with non-communicable diseases.

**Methods:**

A scoping review was conducted by searching PubMed, Embase, Web of Science, and Scopus from January 2000 to May 2024. Eligible studies reported clinical pharmacist interventions in palliative care. Data were extracted on study characteristics, pharmacist activities, and clinical outcomes.

**Results:**

Twelve studies were included, predominantly from the United States. Pharmacist-led interventions encompassed medication reconciliation (91.7%), symptom management (83.3%), adverse drug event prevention (75.0%), patient and caregiver education (58.3%), and policy-level contributions (33.3%). High physician acceptance rates (≥90%) were consistently reported. Outcomes included improved symptom control, reduced drug-related problems, and enhanced patient-reported quality of life.

**Significance of results:**

This scoping review synthesizes current evidence on the roles of clinical pharmacists in palliative care teams. The findings highlight their essential contributions to medication safety, symptom management, deprescribing, and opioid stewardship, reinforcing the need for pharmacist integration into multidisciplinary palliative care models to improve patient-centered outcomes. Future research should focus on implementation models, cost-effectiveness analyses, and service expansion in community-based settings.

## Introduction

Palliative care is defined as active, holistic care for patients whose diseases are no longer responsive to curative treatment, aiming to improve the quality of life for both patients and their families by relieving physical symptoms and addressing psychosocial, emotional, and spiritual needs (Topoll and Arnold 2021; Schnitzer et al. 2023). With the increasing global burden of non-communicable diseases (NCDs), which now account for approximately 71% of all deaths worldwide, the demand for high-quality palliative care services is rapidly growing (Caperon et al. 2018; Janssen et al. 2019). Major contributors include cancer, cardiovascular diseases, chronic respiratory diseases, diabetes, ischemic heart disease, and chronic obstructive pulmonary disease (COPD) (Munday et al. 2019; Namisango et al. 2023).

The cornerstone of palliative care is its multidisciplinary team approach, which integrates various healthcare professionals such as physicians, nurses, pharmacists, social workers, and others (Borgstrom et al. 2025; Venturin et al. 2025). Clinical pharmacists, in particular, are playing an increasingly vital role within palliative care teams (PCTs). The 2020 Guidelines for Rational Use of Palliative and Palliative Care emphasize the essential role of clinical pharmacists in optimizing palliative care. This importance is accentuated by the challenges posed by polypharmacy in patients with NCDs and the reduced physical function that leads to decreased immunity (Monaco et al. 2020). Medication management in palliative care is influenced by several factors, including diminished drug-metabolizing capacity, a higher incidence of drug–drug/food interactions, and an increased risk of adverse drug events (ADEs) (Kuruvilla et al. 2018; Chess-Williams et al. 2024). In response to these complex challenges, clinical pharmacists are ideally positioned to deliver comprehensive medication management to patients with NCDs requiring palliative care. Their expertise allows them to navigate the intricacies of medication regimens in this patient population, significantly contributing to the success of palliative care interventions.

Despite the growing recognition of clinical pharmacists’ value in palliative care, published evidence on their specific roles, interventions, and clinical outcomes remains relatively limited. This scoping review aims to systematically map, summarize, and evaluate the available literature on the clinical activities and professional contributions of pharmacists in PCTs. By identifying patterns in practice and key operational models, this review seeks to clarify the clinical pharmacist’s essential functions in this setting and provide a framework for future research and practice enhancement.

## Materials and methods

### Data source and search strategy

This scoping review was conducted following the PRISMA-ScR (Preferred Reporting Items for Systematic Reviews and Meta-Analyses Extension for Scoping Reviews) guideline (Supplementary File 1). A comprehensive literature search was performed in PubMed, Embase, Web of Science, and Scopus covering publications from January 2000 to May 2024. Full search strings, including MeSH terms, keywords, Boolean operators, and applied filters, were presented in Supplementary File 2. The primary search strategy used combinations of terms related to “Pharmacists,” “Pharmaceutical Services,” and “Palliative Care,” such as (“Pharmacist” AND “Palliative care”) and (“Palliative care” AND “Pharmaceutical services”). Searches were restricted to English-language publications. Gray literature, theses, conference abstracts, and non–peer-reviewed materials were excluded to ensure that only methodologically verifiable evidence was synthesized.

### Data selection

Eligible studies included original research, case reports, or observational studies describing clinical practices and outcomes of pharmacists involved in palliative care for NCD patients. Studies were excluded if they (i) did not explicitly describe pharmacists’ interventions, (ii) were reviews, questionnaire surveys, editorials, or opinion pieces, or (iii) evaluated services provided solely by other healthcare professionals without pharmacist involvement.

Two independent reviewers screened all titles, abstracts, and full texts using EndNote X9.2. Discrepancies were resolved through discussion and consensus with all authors.

### Synthesis of results

Data from included studies were extracted into an Excel spreadsheet and categorized according to publication year, country, type of publication, patient characteristics, pharmacist activities, and reported outcomes.

## Results

### Characteristics of the included studies

**Figure 1.**
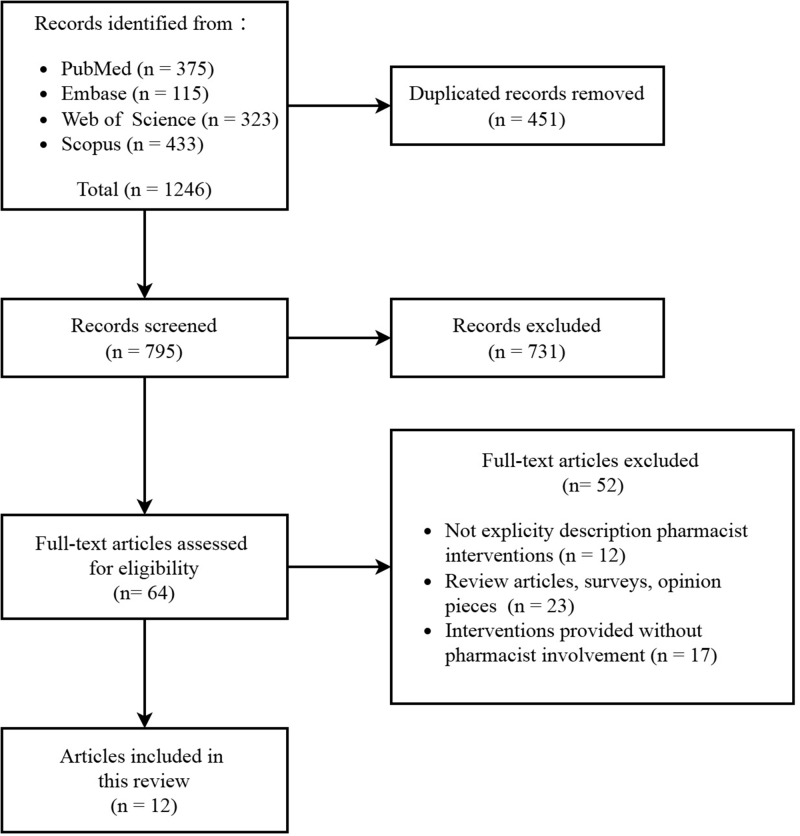
Flowchart of articles identified in scoping review.

The electronic search found 1246 potentially relevant studies. After removing duplicates and reviewing the titles and abstracts, 64 articles were selected for full-text reading. After careful full-text reading, only 12 studies published between the years 2008 and 2024 met the inclusion criteria and thus were included in the review. The flowchart illustrating the search process and reasons for exclusion is shown in Figure 1.

The characteristics of these studies included in this scoping review are summarized in Table 1. The majority of studies were conducted in the United States (n = 8, 66.7%), followed by Canada (n = 2, 16.7%), Thailand (n = 1, 8.3%), and Japan (n = 1, 8.3%). Regarding study design, most publications were observational research articles (n = 8, 66.7%), including retrospective analyses, case series, and descriptive studies, while the remaining were case reports and randomized controlled trials.

**Table 1.** Characteristics of the studies included in scoping review

No.	Author, year	Country	Publication type	Number of patients	Gender and age of patients	Patient characteristics	Purpose of the study
1	Sakthong et al. 2024	Thailand	Research article	120	82 female, 38 male; Mean age: 59.7 (50–69) years	Cancer outpatients with solid tumors receiving anticancer drugs in the chemotherapy unit	To compare the sensitivity of PROMPT-QoL and FACT-G in response to pharmacist-led pharmaceutical care for cancer outpatients
2	Atayee and Edmonds 2023	USA	Case report	4	3 female, 1 male; Age range: 43–63 years	Patients with advanced malignancies or complex palliative needs	To illustrate the roles and entrustable professional activities of palliative care pharmacists within interdisciplinary teams
3	Atayee RS et al., 2018	USA	Brief report	341	209 female, 132 male; Mean age: 53.9 years	Adult hospitalized palliative care patients	To analyze patterns of inpatient palliative care pharmacist interventions and assess their impact on hospital metrics
4	Discala et al. 2017	USA	Research article	11	2 female, 9 male; Mean age: 69.4 (58–93) years	Patients with lung, prostate cancer, and comorbidities including depression, dyspnea, and anxiety	To assess whether an interdisciplinary outpatient palliative clinic improved symptom management outcomes
5	Ma et al. 2016	USA	Original article	80	44 female, 36 male; Mean age: 51.2 (37–65) years	Cancer patients referred for outpatient palliative care	To evaluate pharmacist interventions and outcomes in an outpatient palliative care setting
6	Edwards SJ et al., 2014	Canada	Research article	200	139 female, 61 male; Mean age: 57.1 years	Cancer patients undergoing parenteral chemotherapy	To assess the role of a pharmacist-directed seamless care program on drug-related problems and service satisfaction
7	Gagnon et al. 2012	Canada	Original article	114	37 female, 77 male; Mean age: 68.3 years	Patients receiving palliative radiotherapy for prostate, lung, or breast cancer	To describe the clinical pharmacist’s role in outpatient palliative radiotherapy clinics
8	Wilson S et al., 2011	USA	Research article	117	117 male; Mean age: 75.5 (60–89) years	Patients with various symptoms requiring palliative management	To examine factors influencing physician acceptance of pharmacist recommendations and their clinical outcomes
9	Pituskin E et al., 2010	USA	Research article	82	2 female, 9 male; Mean age: 53.9 (44–88) years	Patients with bone metastases undergoing palliative radiotherapy	To evaluate the feasibility of multidisciplinary palliative assessment in an outpatient radiotherapy setting
10	Fan T et al., 2008	USA	Research article	1744	Not reported	Patients with acute and chronic pain receiving patient-controlled analgesia (PCA)	To report on the implementation and impact of a pharmacy-managed pain service
11	Atayee RS et al., 2008	USA	Research article	29	Not specified; Mean age: 49 (20–78) years	Patients with hematologic malignancies and solid tumors	To develop a model integrating clinical pharmacists into multidisciplinary palliative care teams
12	Myotoku M et al., 2008	Japan	Research article	271	121 female, 150 male; Mean age: 66.9 (46–79) years	Terminal cancer patients initiating opioid therapy	To assess palliative care team activities and medication management during opioid initiation

Most studies (n = 7, 58.3%) were conducted in inpatient or outpatient palliative care settings, while 33.3% (n = 4) focused on outpatient palliative radiotherapy or oncology clinics. Patient numbers varied substantially, from 4 to 1744, with most studies enrolling between 50 and 350 participants. Cancer patients predominated in 10 studies (83.3%), while some included patients with heart failure, advanced COPD, or other terminal NCDs.

Clinical outcomes assessed included symptom control (100%), reduction of drug-related problems (DRPs) (n = 10, 83.3%), improved health-related quality of life (n = 5, 41.7%), medication adherence (n = 4, 33.3%), and health system outcomes such as reduced length of stay or improved discharge rates (n = 3, 25.0%). High physician acceptance rates for pharmacist recommendations, typically exceeding 90%, were reported in several studies.

### Summary of the results of the included studies

Table 2 summarizes the palliative care activities provided by clinical pharmacists. The included studies described a wide range of pharmacist-led interventions, which could be broadly categorized into 5 domains: medication reconciliation (n = 11, 91.7%), symptom management (n = 10, 83.3%), ADE prevention (n = 9, 75.0%), patient and caregiver education (n = 7, 58.3%), and administrative/system-level contributions (n = 4, 33.3%).

**Table 2.** Summary of clinical pharmacist interventions in palliative care settings

No.	Author, year	Pharmacist activities	Key outcomes
1	Sakthong et al. 2024	Medication review, identification of drug-related problems (DRPs), symptom management advice, physician consultation, patient counseling on chemotherapy-related adverse events	PROMPT-QoL was more sensitive in detecting HRQoL improvements with pharmacist-led interventions
2	Atayee and Edmonds 2023	Pharmacotherapy consultation, symptom control, deprescribing, withdrawal management, interdisciplinary care alignment with patient/family values	Clinical cases showed significant symptom relief and improved quality of life; opioid reduction achieved
3	Atayee RS et al., 2018	Patient/family education, healthcare staff training, medication regimen optimization	Early pharmacist involvement improved length of stay, time to consult, and consult-to-discharge intervals
4	Discala et al. 2017	Medication reconciliation, DRP resolution, allergy confirmation, medication adherence assessment, patient education	The MDT team that included clinical pharmacists improved the symptom assessment and palliative care outcomes for patients with advanced cancer and addressed the issue of drug prescriptions.
5	Ma et al. 2016	Medication assessment at multiple outpatient visits, pain score monitoring, medication adjustment	Significant improvement in pain, nausea, and constipation management through pharmacist interventions
6	Edwards SJ et al., 2014	Medication history taking, reconciliation, chemotherapy order review, DRP resolution, patient counseling, telephone follow-up	Improved medication safety, reduced DRPs, enhanced patient outcomes in oncology outpatients
7	Gagnon et al. 2012	Pain history, medication history, opioid toxicity screening, symptom identification, pre-radiotherapy medication management	Pharmacist interventions optimized symptom control, prevented adverse events, and improved interprofessional coordination
8	Wilson S et al., 2011	Pharmacotherapeutic intervention tracking, clinical documentation, recommendation assessment	Acceptance of pharmacist recommendations significantly predicted achievement of clinical outcomes
9	Pituskin E et al., 2010	Opioid toxicity monitoring, supportive medication suggestions, drug interaction checks, medication review	Notable symptom improvement in pain, fatigue, anxiety, and well-being after pharmacist involvement
10	Fan T et al., 2008	Daily PCA patient list review, rounds, initial evaluation within 24 hours, intervention documentation, patient education	Successful implementation of a pain management program with increased PCA utilization over 8 years
11	Atayee RS et al., 2008	Extended patient time, pain/symptom management, psychosocial support	98% of pharmacist recommendations accepted by oncology teams
12	Myotoku M et al., 2008	Prescription design, cancer pain management guidance, opioid adverse effect management, staff education	Improved opioid adverse effect control and quality of life for terminal cancer patients

Medication reconciliation and pharmacotherapy review involved identifying DRPs, optimizing analgesic regimens, adjusting doses for organ dysfunction, deprescribing inappropriate medications, and ensuring safe transitions of care. This was a core intervention in 11 of the 12 studies. Symptom management interventions, especially for pain, dyspnea, nausea, and constipation, were reported in 10 studies (83.3%).

Adverse event prevention, including proactive monitoring of opioid-induced side effects, opioid toxicity, QTc prolongation, and cumulative sedative load, was addressed in 9 studies. Pharmacists also provided structured patient and family education in 7 studies (58.3%), covering medication regimens, opioid safety, symptom management, and deprescribing plans.

Administrative contributions, such as formulary management, opioid stewardship, and institutional guideline development, were described in 4 studies. Three studies (25.0%) documented pharmacist involvement in healthcare professional training, reflecting growing emphasis on interdisciplinary education.

Overall, interventions demonstrated consistently high physician acceptance rates (≥90%) and contributed to improved symptom control, enhanced patient satisfaction, reduced DRPs, and optimized medication use across both inpatient and outpatient palliative care services.

## Discussion

### Scoping of the current evidence

This scoping review comprehensively examined the evolving roles and clinical practices of pharmacists within multidisciplinary PCTs across diverse healthcare settings. The findings confirm that clinical pharmacists provide indispensable contributions to optimizing pharmacotherapy, enhancing medication safety, and improving quality of life for patients with advanced NCDs. Pharmacists consistently demonstrated expertise in medication reconciliation, DRP resolution, adverse event prevention, symptom management, and multidisciplinary clinical decision-making.

The evidence suggests that pharmacist-led pharmaceutical care significantly improves patient-reported outcomes (PROs) and facilitates individualized, goal-concordant pharmacotherapy. As shown by Sakthong et al. (2024), pharmacist interventions enhanced health-related quality of life for cancer outpatients through proactive identification and resolution of medication-related issues. Similarly, Atayee and Edmonds (2023) highlighted pharmacists’ pivotal role in complex symptom control, opioid rotation, deprescribing, and family-centered care discussions for end-of-life patients. Studies consistently reported high acceptance rates of pharmacist recommendations, reaching over 90% in some outpatient and inpatient programs (Gagnon et al. 2012; Ma et al. 2016; Discala et al. 2017), reflecting their essential integration within multidisciplinary palliative care services.

Moreover, the studies included in this review highlight the broadening scope of pharmacist responsibilities in palliative care. Their contributions extend to pain and symptom management, support for mental health concerns, nutritional guidance, adherence monitoring, and involvement in advance care planning. Pharmacists also led patient and caregiver education, healthcare professional training, research initiatives, formulary management, and opioid stewardship programs, directly contributing to institutional medication safety and quality improvement frameworks (Franco et al. 2022; Geiger et al. 2022; Wernli et al. 2023). These findings collectively demonstrate that clinical pharmacists are not merely auxiliary members of PCTs, but central contributors to comprehensive, patient-centered, and evidence-based palliative care delivery.

Despite the valuable insights provided, the predominance of studies originating from the United States may influence the generalizability of the findings. Palliative pharmacy services in the United States often benefit from well-established interdisciplinary structures, clear reimbursement pathways, and defined clinical pharmacist roles. These features may differ substantially from those in low-resource environments or healthcare systems with distinct regulatory, financial, or workforce characteristics. Consequently, models of pharmacist engagement that require specialized training or intensive staffing may not be directly transferable to all regions. Future research conducted in geographically and economically diverse settings is needed to strengthen the global evidence base and support the development of context-appropriate frameworks for palliative pharmacy practice.

### Role of palliative care pharmacists

#### Pharmacist-led symptom management optimization

Effective symptom control remains a principal objective in palliative care, with clinical pharmacists assuming a critical role in optimizing pharmacotherapy for distressing symptoms such as pain, dyspnea, nausea, constipation, delirium, and anxiety. Pharmacist contributions include evidence-based medication selection, dose adjustments, opioid titration and rotation, adjuvant therapy recommendations, and breakthrough pain protocol development. Gagnon et al. (2012) demonstrated that integrating pharmacists into outpatient radiotherapy palliative care services significantly improved analgesic prescribing patterns, symptom relief, and reduced the frequency of uncontrolled breakthrough pain episodes.

Beyond analgesia, pharmacists contribute to the management of nausea, vomiting, and opioid-induced constipation by selecting appropriate agents, adjusting doses based on hepatic and renal function, and implementing prophylactic regimens for high-risk adverse effects. Ma et al. (2016) highlighted that in ambulatory palliative care settings, pharmacist interventions addressing antiemetics, laxatives, and anxiolytics represented a significant proportion of clinical recommendations, directly improving symptom control and patient tolerability. Recent prospective evidence by Sakthong et al. (2024) confirmed that pharmacist-led pharmaceutical care significantly improved patient-reported quality of life scores and reduced medication-related problems in cancer outpatients receiving palliative care. Furthermore, Rabow et al. (2017) and Yates (2017) underscored the importance of structured multidisciplinary approaches, integrating pharmacists to enhance symptom burden reduction and improve health-related quality of life in advanced disease populations.

In frail elderly and cognitively impaired patients, the challenge of symptom management is further amplified by altered pharmacokinetics and heightened adverse event risk. Drummond and Johnston (2024) emphasized that pharmacist-led tailored pharmacotherapy adjustments for agitation, pain, and respiratory symptoms at end-of-life substantially improved patient comfort and reduced polypharmacy-associated complications. Importantly, the expansion of home-based and outpatient palliative care models has further demonstrated the value of pharmacists in remote medication consultation, symptom monitoring, and telehealth-based pharmacotherapy adjustments, ensuring continuity and safety of care beyond hospital environments.

#### Medication reconciliation and comprehensive pharmacotherapy review

Medication reconciliation is a fundamental component of pharmacotherapy safety in palliative care, given the high prevalence of polypharmacy, multimorbidity, and the frequent adjustment of medication regimens in this population. At critical points of care transition, including hospital admission, interdepartmental transfer, discharge, and hospice referral, clinical pharmacists play an indispensable role in ensuring the accuracy, appropriateness, and clinical relevance of medication lists. Through comprehensive medication reviews, pharmacists systematically identify DRPs, potential drug–drug and drug–disease interactions, instances of inappropriate polypharmacy, and opportunities for deprescribing based on prognosis and patient-centered care objectives.

Multiple studies have substantiated the clinical value of pharmacist-led medication reconciliation services. A systematic review and meta-analysis by Mekonnen et al. (2016) demonstrated that reconciliation interventions conducted by pharmacists effectively reduced medication discrepancies, ADEs, and hospital readmissions in acutely ill patients – outcomes highly pertinent to palliative care populations. Consistent with these findings, Berthe et al. (2017) reported in a prospective study of elderly inpatients that structured medication reconciliation by clinical pharmacists markedly decreased medication errors and potential ADEs, particularly among cognitively impaired individuals with complex comorbidities, a scenario frequently encountered in palliative care settings. Further confirmation comes from a Cochrane review by Redmond et al. (2018), which concluded that pharmacist involvement in medication reconciliation at care transitions substantially reduced clinically significant medication discrepancies and associated harms. Collectively, these data affirm the essential role of pharmacists in improving medication safety, supporting symptom management, and ensuring pharmacotherapy remains aligned with patients’ disease progression and individualized care goals.

Given the dynamic nature of palliative pharmacotherapy, where treatment regimens frequently shift from disease-modifying to symptom-directed therapies, pharmacist-led medication reconciliation is crucial not only for minimizing harm but also for simplifying regimens, enhancing adherence, and improving patients’ quality of life. Integrating these services into routine palliative care workflows is increasingly advocated in international patient safety guidelines.

#### Adverse drug reaction monitoring and prevention

Palliative care patients are inherently at high risk for adverse drug reactions (ADRs) due to polypharmacy, advanced age, organ dysfunction, frailty, and the frequent use of high-risk medications, including opioids, benzodiazepines, antipsychotics, and corticosteroids. Within multidisciplinary PCTs, clinical pharmacists are indispensable in proactively monitoring, preventing, and managing ADRs through comprehensive medication reviews and individualized risk assessments.

Pharmacists participate directly in multidisciplinary discussions, offering real-time pharmacotherapy reviews and implementing structured ADR monitoring protocols tailored to each patient’s clinical status and medication profile. Mann et al. (2018) demonstrated in hospital-at-home programs that over 30% of patients experienced at least 1 medication-related problem, highlighting the need for pharmacist-led pharmacovigilance interventions even in community palliative care. Among opioid-related ADRs, respiratory depression represents a particularly serious risk. Sugawara et al. (2023) used a large national pharmacovigilance database to emphasize the importance of vigilant opioid monitoring in both cancer and non-cancer palliative populations, recommending pharmacist oversight for titration protocols and early detection strategies.

In the evolving landscape of personalized palliative care, Silva et al. (2024) advocated for integrating precision pharmacovigilance approaches, incorporating pharmacogenetic data and individualized risk prediction models into routine pharmacotherapy, with pharmacists ideally positioned to lead these innovations. Additionally, incorporating PROs into routine ADR monitoring enhances the sensitivity of symptom-based toxicity detection. Albaba et al. (2019) demonstrated that oncology outpatients benefited from PRO-CTCAE tools when clinical pharmacists led medication reviews and multidisciplinary discussions attributing symptoms to ADRs.

Beyond direct pharmacovigilance, pharmacists play key roles in opioid stewardship initiatives, deprescribing audits, and polypharmacy management programs. As Franco et al. (2022) and Geiger et al. (2022) noted, pharmacists assume leadership in developing institutional protocols for managing high-risk medications, formulary oversight, and clinician education in safe prescribing practices. These contributions reinforce the indispensable role of clinical pharmacists in improving medication safety, reducing preventable hospitalizations, and enhancing the overall quality and safety of end-of-life care.

#### Deprescribing and individualized pharmacotherapy adjustment

As patients with life-limiting illnesses progress, many chronic medications become clinically unnecessary or potentially harmful. Deprescribing is a structured, patient-centered process for discontinuing unnecessary or inappropriate medications and represents a core competency of clinical pharmacists in palliative care. Through systematic medication reviews, pharmacists assess the appropriateness of ongoing treatments, taking into account prognosis, symptom burden, drug–disease interactions, and the patient’s evolving goals of care.

Kuruvilla et al. (2018) highlighted the pivotal role of specialist palliative care pharmacists in discontinuing non-beneficial medications, such as cardiovascular preventives (including statins, antihypertensives, and antiplatelets), hypoglycemics, psychotropics, and dietary supplements, particularly in community-based settings. These interventions ensure medication regimens remain clinically appropriate, tolerable, and congruent with patient preferences. In parallel, pharmacists frequently adjust pharmacotherapy in response to rapid changes in organ function, drug absorption, cognitive status, and overall physiological reserve commonly encountered in late-stage disease.

Emerging evidence from pharmacist-led deprescribing initiatives supports the clinical value of these interventions. Green et al. (2024) piloted a telehealth-based deprescribing program targeting elderly dementia patients with polypharmacy, achieving meaningful reductions in medication burden and improvements in symptom control, demonstrating its feasibility for home-based palliative care. Similarly, Jovevski et al. (2023) implemented a mandatory pharmacist-led deprescribing intervention for high-risk emergency department patients, significantly reducing inappropriate medication use and ADE risk, affirming the practicality of pharmacist-driven deprescribing in acute care transitions. High-quality randomized trial evidence also supports pharmacist deprescribing leadership. The D-Prescribe trial by Martin et al. (2018) demonstrated that a pharmacist-led educational intervention significantly reduced inappropriate medication use in older adults, with sustained deprescribing effects over time. Collectively, these findings highlight the dual role of deprescribing in medication optimization and in improving patient quality of life, reducing symptom burden, and minimizing the risks associated with polypharmacy.

Within multidisciplinary PCTs, clinical pharmacists are uniquely positioned to lead these deprescribing processes in close collaboration with physicians, nurses, and patients. Their responsibilities extend beyond discontinuing medications to encompass individualized pharmacotherapy adjustments, including careful dose tapering of opioids, benzodiazepines, and antipsychotics. Such adjustments are critical to preventing withdrawal symptoms and ensuring safe, symptom-focused transitions in end-of-life care.

#### Education of patient, caregiver, and healthcare professionals

Education remains a fundamental and indispensable responsibility of clinical pharmacists within multidisciplinary PCTs. Pharmacists are uniquely positioned to provide comprehensive, patient-centered education to patients and their caregivers, covering essential topics such as medication administration techniques, opioid safety precautions, breakthrough pain management protocols, recognition and management of adverse drug effects, and the proper storage and disposal of controlled substances. This role is particularly critical in home-based palliative care settings, where patients and families assume greater responsibility for daily medication management. Tait et al. (2020) reported that pharmacist-led counseling interventions for community-dwelling palliative care patients and their families effectively improved medication adherence, reduced opioid misuse risks, and enhanced caregiver confidence in managing complex pharmacotherapy regimens.

In the hospital and hospice settings, pharmacists also assume leadership roles in educating healthcare professionals, including physicians, nurses, and allied health staff. Through formal in-service training, case-based teaching, and multidisciplinary rounds, pharmacists enhance team members’ knowledge of complex pharmacotherapy issues, opioid stewardship, ADR management, and deprescribing principles. Moody et al. (2022) demonstrated that structured educational programs led by clinical pharmacists significantly improved clinician competencies in opioid rotation, opioid equivalency conversions, and safe deprescribing practices within hospice palliative care settings. Further reinforcing this, Atayee and Edmonds (2023) illustrated how specialist palliative care pharmacists actively participate in multidisciplinary decision-making meetings, providing real-time pharmacotherapy consultations, medication safety education, and anticipatory guidance for symptom control protocols. Geiger et al. (2022) emphasized the expanding educational responsibilities of clinical pharmacists in palliative care, noting their critical leadership roles in training program development, mentorship of junior pharmacists and clinicians, and evidence-based policy formulation initiatives within institutional palliative care services.

Moreover, patient and caregiver education has been directly associated with improved clinical outcomes in palliative care settings. Elkhateeb and Salem (2018) found that higher levels of patient and family medication literacy were correlated with reduced rates of hospital readmission and mortality in heart failure patients receiving palliative services. This evidence suggests similar potential benefits of pharmacist-led educational interventions in cancer palliative care and advanced chronic illness populations, reinforcing the importance of pharmacist involvement in education as a core dimension of comprehensive palliative care practice.

#### Contribution to policy, formulary management, and opioid stewardship

As core members of Pharmacy and Therapeutics (P&T) committees and multidisciplinary governance structures, pharmacists provide critical input on essential drug lists, opioid formulary standards, and deprescribing guidelines, ensuring these frameworks address the complex, rapidly evolving needs of palliative care populations. Accumulating evidence confirms that pharmacist-led opioid stewardship initiatives substantially improve prescribing appropriateness, reduce dosing errors, and enhance risk mitigation practices, particularly in older adults and home-based care. Interventions such as dose optimization, adverse effect prophylaxis, opioid misuse prevention, and protocol adherence have demonstrated consistent clinical benefits across care settings (Mohammad et al. 2024; Shrestha et al. 2025). Furthermore, Kral et al. (2022) demonstrated that palliative care pharmacists frequently contribute to institutional opioid tapering policies and rotation protocols, reflecting their expanding authority in stewardship policy development and controlled substance governance. This leadership function has been formally codified by the Society of Pain and Palliative Care Pharmacists, with DiScala et al. (2023) defining standardized competencies for pharmacists in opioid policy drafting, prescribing audits, and the development of institution-specific pathways for breakthrough pain and opioid-related adverse effect management.

Beyond opioid governance, pharmacists oversee the selection, review, and formulary management of essential palliative medications, including adjunctive therapies such as antiemetics, anxiolytics, corticosteroids, and palliative sedation agents (Geiger et al. 2022; Moody et al. 2022). Notably, Hill et al. (2022) demonstrated that pharmacist-led formulary optimization and stewardship initiatives not only improved clinical outcomes but also reduced healthcare expenditures, further substantiating the operational and economic value of pharmacist integration in palliative care governance.

Collectively, these policy-driven and operational responsibilities position clinical pharmacists as indispensable stewards of pharmacotherapy safety, regulatory compliance, and therapeutic equity within contemporary palliative care services. Their leadership ensures that medication policies remain responsive to the complex, shifting needs of palliative populations while upholding the highest standards of patient safety and quality of care.

### Key points and challenges for palliative care pharmacists

The clinical management of palliative care patients has become increasingly complex, driven by multimorbidity, frailty, progressive organ dysfunction, and highly variable pharmacokinetic profiles. This complexity demands individualized, dynamic pharmacotherapy oversight, requiring clinical pharmacists to exercise advanced clinical judgment, nuanced understanding of end-of-life symptomatology, and excellent interdisciplinary communication skills to optimize medication use for this vulnerable population.

One of the foremost challenges lies in the selection, titration, and deprescribing of high-risk medications, including opioids, benzodiazepines, antipsychotics, and corticosteroids. These agents are routinely used in palliative care, often in patients with fluctuating organ function and multiple comorbidities. Pharmacists must therefore possess advanced pharmacokinetic knowledge and deprescribing competencies to safely adjust therapies while minimizing cumulative sedative, anticholinergic, and delirium risks. Wernli et al. (2023) documented that clinical pharmacists in inpatient hospice and palliative care routinely conduct complex medication reviews, manage opioid conversions, and address multiple DRPs per patient, underscoring their essential role in medication safety management.

Effective, empathetic communication remains a central pharmacist’s responsibility within PCTs. Uritsky et al. (2018) emphasized that pharmacists must be skilled in navigating sensitive discussions with patients and families regarding medication benefits, adverse effects, deprescribing decisions, and end-of-life medication plans. This requires not only pharmacotherapeutic expertise but also advanced psychosocial counseling abilities tailored to highly emotive, end-of-life care contexts.

Operational constraints continue to challenge service provision globally. Doobay-Persaud et al. (2023) identified severe workforce shortages and medication accessibility barriers as major limitations in resource-limited settings, with similar under-resourcing of pharmacist positions reported even within well-established palliative care services in developed healthcare systems. To address this, the development of sustainable clinical service models and the formal integration of pharmacists into multidisciplinary teams are urgently needed to support their expanding clinical, educational, and operational leadership responsibilities.

As the discipline advances, continuous professional development remains critical, particularly in emerging domains such as deprescribing frameworks and opioid stewardship leadership. Makki and Bartell et al. (2025) demonstrated the feasibility of implementing a pharmacist-led, standardized deprescribing assessment model within specialist palliative care services, improving medication appropriateness while minimizing unnecessary drug burden. Building on this, McAdam et al. (2025) provided compelling evidence for the clinical impact of structured pharmacist-led deprescribing initiatives in cancer palliative care. Their study incorporated validated palliative care assessment tools, including the STOPPFrail criteria and Palliative Prognostic Index, into a pharmacist-driven workflow, resulting in improved medication appropriateness, enhanced symptom control, and reduced ADEs. Notably, this model offers a replicable operational framework for future real-world implementation research evaluating deprescribing feasibility, multidisciplinary team acceptance, and patient- and caregiver-reported outcomes across diverse care settings.

With the continued expansion of home-based and community palliative care models, pharmacists face new clinical, psychosocial, and operational demands. Wu et al. (2024) described the professional role-transition challenges experienced by community pharmacists integrating into home-based palliative services, highlighting the necessity for dedicated training programs, interprofessional coordination, and structured support strategies to facilitate their adaptation to complex end-of-life care environments.

In alignment with their expanding responsibilities, palliative care pharmacists are increasingly recognized as indispensable members of multidisciplinary teams, contributing expertise in symptom control optimization, medication safety, opioid stewardship, and formulary policy development (Franco et al. 2022; Geiger et al. 2022). Looking ahead, the integration of digital pharmaceutical services provides a promising avenue to address many of the challenges identified in this review. Innovations such as telepharmacy and artificial intelligence (AI)-assisted medication management can extend pharmacists’ reach, particularly in community or home-based care. AI-driven clinical decision support tools can analyze electronic health data to detect drug interactions, predict adverse events, and identify deprescribing opportunities, thereby enhancing the efficiency of pharmacist-led interventions. Telepharmacy enables remote consultations, symptom monitoring, and patient education, improving continuity of care for homebound or rural patients. In addition, digital adherence tools, smart pillboxes, and wearable sensors can generate real-time data to support individualized medication management. Although issues such as digital literacy, data security, and equitable access must be addressed, these technologies offer substantial potential to support personalized, proactive, and scalable palliative pharmacy services. Future research should prioritize the development, implementation, and cost-effectiveness evaluation of these emerging digital care models.

## Conclusions

This scoping review highlights the essential and increasingly complex role of clinical pharmacists within multidisciplinary PCTs. Across diverse care settings, pharmacists have consistently demonstrated their value in optimizing pharmacotherapy, improving symptom control, reducing ADEs, and enhancing health-related quality of life for patients with advanced NCDs. Through comprehensive medication reconciliation, individualized symptom management, adverse event prevention, deprescribing, patient and caregiver education, and contributions to institutional policy and opioid stewardship, clinical pharmacists serve as integral members of palliative care services.

The findings confirm that pharmacist-led interventions achieve high clinical acceptance rates and significantly improve medication safety and patient-centered care outcomes. However, it is important to acknowledge the limitations of this review. The inclusion of studies only published in English may have introduced language bias, and the decision not to search gray literature might have omitted recent initiatives. Furthermore, the review protocol was not prospectively registered on the Open Science Framework, and this has been acknowledged as a methodological limitation. Despite these limitations, the findings provide a robust overview of the current evidence.

Beyond the methodological considerations of this review, several practical challenges remain, including limited staffing resources, inconsistent integration into palliative care frameworks, and the need for ongoing professional development. Future research should focus on establishing standardized models for pharmacist-led palliative care services, conducting rigorous evaluations of their clinical and economic impact, and expanding implementation in community and home-based settings. Strengthening the pharmacist’s role in palliative care holds great potential to advance the quality, safety, and equity of care for patients facing life-limiting illness.

## Supporting information

10.1017/S1478951525101545.sm001 Li et al. supplementary material Li et al. supplementary material

## Supplementary material

The supplementary material for this article can be found at https://doi.org/10.1017/S1478951525101545.
