# Supplementary material for: Role of clinical pharmacists in palliative care team: A scoping review
Source: Palliat Support Care. 2026 Jan 13;24:e33. doi: 10.1017/S1478951525101545 (PMC13166493; doi:10.1017/S1478951525101545)
Supplement: Li et al. supplementary material [file S1478951525101545sup001.docx]

Supplementary Material

**Role of clinical pharmacists in palliative care team: A scoping review**

Li Sen^1#^, Wang Qin^2#^, Qi Benling^3^, Bai Lijuan^3^, Xu Jiaqiang^1^, Sun Haiying^4*^, Liu Yihui^1*^

1. Department of Pharmacy, Union Hospital, Tongji Medical College, Huazhong University of Science and Technology, 430030 Wuhan, Hubei Province, China.

2. Department of Radiology, Union Hospital, Tongji Medical College, Huazhong University of Science and Technology, Wuhan, 430022, China.

3. Department of Geriatrics, Union Hospital, Tongji Medical College, Huazhong University of Science and Technology, Wuhan, Hubei 430022, China.

4. Department of Otorhinolaryngology, Union Hospital, Tongji Medical College, Huazhong University of Science and Technology, Wuhan, 430022, China.

#These authors contributed equally to this work.

*Corresponding author:

Liu Yihui, E-mail: kafkaliu@163.com

Sun Haiying, E-mail: sunhaiying120@hust.edu.cn

**Supplementary File 1. PRISMA-ScR checklist.**

| **SECTION** | **ITEM** | **PRISMA-ScR CHECKLIST ITEM** | **REPORTED**  **ON PAGE #** |
| --- | --- | --- | --- |
| **TITLE** | | | |
| Title | 1 | Identify the report as a scoping review. | Title page |
| **ABSTRACT** | | | |
| Structured  summary | 2 | Provide a structured summary that includes (as applicable): background, objectives, eligibility  criteria, sources of evidence, charting methods, results, and conclusions that relate to the review questions and objectives. | Abstract section |
| **INTRODUCTION** | | | |
| Rationale | 3 | Describe the rationale for the review in the context of what is already known. Explain why the review questions/objectives lend themselves to a scoping review approach. | Introduction |
| Objectives | 4 | Provide an explicit statement of the questions and objectives being addressed with reference to their key elements (e.g., population or participants,  concepts, and context) or other relevant key elements used to conceptualize the review questions and/or objectives. | Introduction |
| **METHODS** | | | |
| Protocol and registration | 5 | Indicate whether a review protocol exists; state if and where it can be accessed (e.g., a Web  address); and if available, provide registration information, including the registration number. | Section 2.1 (not registered; noted as limitation) |
| Eligibility criteria | 6 | Specify characteristics of the sources of evidence used as eligibility criteria (e.g., years considered, language, and publication status), and provide a rationale. | Section 2.2 |
| Information sources* | 7 | Describe all information sources in the search (e.g., databases with dates of coverage and contact with authors to identify additional sources), as well as  the date the most recent search was executed. | Section 2.1 |
| Search | 8 | Present the full electronic search strategy for at least 1 database, including any limits used, such that it could be repeated. | Supplementary File 2 |
| Selection of  sources of evidencet | 9 | State the process for selecting sources of evidence (i.e., screening and eligibility) included in the  scoping review. | Section 2.2 |
| Data charting process | 10 | Describe the methods of charting data from the  included sources of evidence (e.g., calibrated forms or forms that have been tested by the team before their use, and whether data charting was done  independently or in duplicate) and any processes  for obtaining and confirming data from investigators. | Section 2.3 |
| Data items | 11 | List and define all variables for which data were sought and any assumptions and simplifications made. | Section 2.3 |
| Critical appraisal of individual  sources of evidence§ | 12 | If done, provide a rationale for conducting a critical appraisal of included sources of evidence; describe the methods used and how this information was  used in any data synthesis (if appropriate). | Not applicable |

| **SECTION** | **ITEM** | **PRISMA-ScR CHECKLIST ITEM** | **REPORTED**  **ON PAGE #** |
| --- | --- | --- | --- |
| Synthesis of results | 13 | Describe the methods of handling and summarizing the data that were charted. | Section 2.3 |
| **RESULTS** | | | |
| Selection of  sources of evidence | 14 | Give numbers of sources of evidence screened,  assessed for eligibility, and included in the review, with reasons for exclusions at each stage, ideally using a flow diagram. | Figure 1 |
| Characteristics of sources of  evidence | 15 | For each source of evidence, present  characteristics for which data were charted and provide the citations. | Section 3.1 |
| Critical appraisal within sources of evidence | 16 | If done, present data on critical appraisal of included sources of evidence (see item 12). | Not applicable |
| Results of  individual sources of evidence | 17 | For each included source of evidence, present the relevant data that were charted that relate to the  review questions and objectives. | Section 3.2; Table 1 and 2 |
| Synthesis of results | 18 | Summarize and/or present the charting results as they relate to the review questions and objectives. | Table 1 and 2 |
| **DISCUSSION** | | | |
| Summary of evidence | 19 | Summarize the main results (including an overview of concepts, themes, and types of evidence  available), link to the review questions and  objectives, and consider the relevance to key groups. | Section 4 |
| Limitations | 20 | Discuss the limitations of the scoping review process. | Section 5 |
| Conclusions | 21 | Provide a general interpretation of the results with respect to the review questions and objectives, as well as potential implications and/or next steps. | Section 5 |
| **FUNDING** | | | |
| Funding | 22 | Describe sources of funding for the included  sources of evidence, as well as sources of funding for the scoping review. Describe the role of the  funders of the scoping review. | Funding statement |

**Supplementary File 2. Full search strategies for databases.**

**1. PubMed Search Strategy**

Database: PubMed

Coverage: January 2000 – May 2024

Filters applied: English, Humans

Search string:

#1 "Pharmacists"[Mesh] OR pharmacist*[tiab] OR "clinical pharmacist*"[tiab]

#2 "Palliative Care"[Mesh] OR "Hospice and Palliative Care Nursing"[Mesh]

OR "palliative care"[tiab] OR "palliative medicine"[tiab]

OR hospice[tiab] OR "end of life"[tiab]

#3 "Pharmaceutical Services"[Mesh] OR "medication therapy management"[tiab]

OR "medication review"[tiab] OR "pharmacotherapy"[tiab]

#4 #1 AND (#2 OR #3)

Filters: English; Humans; Publication date from 2000/01/01 to 2024/05/31

**2. Embase (Elsevier) Search Strategy**

Database: Embase.com

Coverage: January 2000 – May 2024

Filters applied: English language; Human studies; Articles only

Search string:

('pharmacist'/exp OR pharmacist*:ti,ab OR 'clinical pharmacist*':ti,ab)

AND

('palliative therapy'/exp OR 'palliative care':ti,ab OR

'palliative medicine':ti,ab OR hospice:ti,ab OR 'end of life':ti,ab)

AND

('pharmaceutical care'/exp OR 'medication therapy management':ti,ab OR

'medication review':ti,ab OR pharmacotherapy:ti,ab)

Limits: English; Humans; Article; 2000–2024

**3. Web of Science Core Collection Search Strategy**

Database: WoS Core Collection

Coverage: January 2000 – May 2024

Filters applied: English, Articles

Search string (Topic Search = TS):

TS = (pharmacist* OR "clinical pharmacist*")

AND

TS = ("palliative care" OR "palliative medicine" OR hospice OR "end of life")

AND

TS = ("pharmaceutical care" OR "medication review" OR "medication therapy management"

OR pharmacotherapy)

Refined by: English; Articles; Year 2000–2024

**4. Scopus Search Strategy**

Database: Scopus

Coverage: January 2000 – May 2024

Filters applied: English, Articles

Search string:

(TITLE-ABS-KEY (pharmacist* OR "clinical pharmacist*"))

AND

(TITLE-ABS-KEY ("palliative care" OR "palliative medicine"

OR hospice OR "end of life"))

AND

(TITLE-ABS-KEY ("pharmaceutical care" OR "medication review"

OR "medication therapy management" OR pharmacotherapy))

Limit to: English; Article; 2000–2024
